# “It is not an acceptable disease”: A qualitative study of HIV-related stigma and discrimination and impacts on health and wellbeing for people from ethnically diverse backgrounds in Australia

**DOI:** 10.1186/s12889-021-10679-y

**Published:** 2021-04-23

**Authors:** Anna Ziersch, Moira Walsh, Melanie Baak, Georgia Rowley, Enaam Oudih, Lillian Mwanri

**Affiliations:** 1 College of Medicine and Public Health, Flinders University, Adelaide, Australia; 2 School of Education, University of South Australia, Adelaide, Australia; 3 College of Nursing and Health Sciences, Flinders University, Adelaide, Australia; 4 Relationships Australia South Australia, Adelaide, Australia

**Keywords:** HIV, stigma, discrimination, migrant, refugee, health, ethnically diverse, CALD, Australia

## Abstract

**Background:**

People from ethnically diverse backgrounds living with HIV are susceptible to adverse health and wellbeing outcomes, particularly as a consequence of HIV-related stigma and discrimination (HSD), though relatively little is known about experiences in Australia.

**Methods:**

This paper reports on HSD in ethnically diverse communities in South Australia and impacts on health and wellbeing. Interviews and focus groups were conducted with 10 individuals living with HIV from ethnically diverse backgrounds, 14 ethnically diverse community leaders, and 50 service providers. Data were analysed thematically.

**Results:**

Findings indicated that HIV is a highly stigmatised condition in ethnically diverse communities due to fear of moral judgment and social isolation, and was experienced at the intersections of gender, sexual orientation, religion, culture, and immigration status. Experiences of HSD were damaging to health and wellbeing through non-disclosure, reduced social support, delayed testing, service access barriers, impacts on treatment adherence, and directly to mental health.

**Conclusions:**

Actions addressing the impacts of HSD on people from ethnically diverse backgrounds are crucial.

**Supplementary Information:**

The online version contains supplementary material available at 10.1186/s12889-021-10679-y.

## BACKGROUND

At the end of 2018 almost 38 million people were living with HIV [1]. In Australia, this figure was more than 26,000 [2]. People born outside Australia comprise a third of HIV notifications by heterosexual transmission and were highlighted as a priority population in the latest National HIV Strategy [3]. Despite improvements in attitudes around HIV, HIV-related stigma and associated discrimination (HSD) remain a key challenge for those living with HIV and their communities. However, while more broadly HSD is well-researched, there is relatively little known about the health and wellbeing impacts of HSD within ethnically diverse and migrant communities in Australia, particularly from the perspectives of people from ethnically diverse backgrounds living with HIV. Drawing on interviews and focus groups with people living with HIV (PLHIV), community leaders, and service providers, this study explored experiences of HSD for PLHIV from ethnically diverse communities and impacts of this on health and wellbeing (hereafter 'health').

We recognise the use of terminology to describe groups of people based on ethnic and migrant background is a cause of debate. In Australia, the generally accepted phrasing is ‘Culturally and Linguistically Diverse’ (CALD) communities or background. The Australian Bureau of Statistics (ABS) defines the CALD population as people from a range of different countries or ethnic and cultural groups according to country of birth and year of arrival in Australia, parents’ country of birth, language spoken at home, English proficiency, and religious affiliation [4]. Given the Australian focus of this empirical study and the Australian organisations we worked with, we therefore use the term ‘CALD’ throughout the rest of the paper.

### Stigma

Stigma refers to a devaluing social identity which casts certain characteristics and behaviours as socially undesirable [5, 6]. As a social process, stigma reveals itself at the structural level through the presence of unequal power relations in social norms, systems and institutions that result in discrimination and status loss for those who transgress valued social identities [6]. Stigma is also perceived, enacted and reinforced through local and cultural norms and values at the community level in the context of relationships with family, friends, intimate partners at the interpersonal level. Moreover, individual level stigma operates through: experienced or enacted stigma (actual experiences of discrimination, prejudice or devaluation by others); perceived stigma (an individual’s perceptions of stigmatising attitudes that may be present in the community); anticipated stigma (an individual’s expectations of poor treatment by others), and internalised stigma (the devaluing of oneself or the group one belongs to with associated feelings and cognitions of embarrassment and shame )[7, 8]. Importantly, the various levels of stigma processes described here are interdependent and bi-directional in so far as stigma at a structural level forms community and individual processes, and visa-versa.

### HIV-related stigma and associated discrimination, and health and wellbeing

According to UNAIDS, HIV-related stigma “refers to the negative beliefs, feelings and attitudes towards people living with HIV, groups associated with people living with HIV and other key populations at higher risk of HIV infection” and HIV-related discrimination is “the unfair and unjust treatment (act or omission) of an individual based on his or her real or perceived HIV status” [9](p.g.2). For many PLHIV, HSD is a significant health issue [10–13], with reviews indicating HSD poses a range of negative physical and mental health impacts [11, 14, 15]. Specifically, these studies have noted that perceived and experienced HSD negatively impact service utilisation including testing and antiretroviral therapy uptake and adherence, subsequently impacting on physical health. Moreover, HSD can have a deleterious effect on mental health, which further impacts disclosure, social interactions and quality of life [11]. HSD has also been identified as compromising the quality of care provided to people living with HIV, their partners, and communities [16–18]. Furthermore, self-stigma is particularly harmful to health because the vigilance required to anticipate and manage such treatment becomes a psychosocial stressor [5, 11], which can persist even when discriminatory treatment is not experienced [19].

Research originating from the African Region, which accounts for almost two thirds of the global total of new HIV infections [20] and the origin of a significant population of Australia’s CALD communities via refugee resettlement pathways, details key misconceptions influenced by ethnicity, gender, region and religion in relation to HIV transmission. These misconceptions contribute to fear and panic and heightened vulnerability to transmission of the disease, and likely contribute to HSD in resettlement countries [21–23].

### HIV stigma and health for people from CALD backgrounds

Research has examined HIV stigma for people from CALD backgrounds and found significant levels of HSD in high income countries such as Canada, the United Kingdom, the United States, and the Netherlands with most studies reporting on Sub-Saharan African and Caribbean diaspora communities [24–27]. Socio-cultural factors that associate HIV with death, immorality, promiscuity and sexually ‘deviant’ behaviours and the fear of contagion, were often reinforced by religious beliefs [28–31].

Specifically, HSD has been identified as a significant barrier to HIV testing [24, 25, 32, 33], health service access [29, 34–37], reduced engagement in preventive approaches [32] and treatment adherence [38]. Fear of social rejection and isolation from communities has also been identified [25, 26, 33]. While most studies found PLHIV chose to keep their positive status a secret to avoid stigma, Koku [39] found a small number of individuals attempted to confront stigma and normalised HIV through public disclosure.

There is an emerging consideration of how PLHIV from CALD backgrounds may experience multiple forms of stigma [36, 40] where HSD is compounded by a range of factors such as race, gender, social and economic disadvantage [29, 31, 35]. For migrant populations, particularly those from refugee backgrounds, these multiplicative stigmas can further interact with aspects of integration such as language barriers, housing, employment, immigration and legal status and anti-asylum discourses [27, 29]. Additionally, multiple stigmatisation has been shown to exacerbate risk of transmission, contribute to prejudicial treatment in healthcare settings, and negatively affect access to prevention and treatment services and survival [10, 36, 37, 41, 42].

In Australia, limited studies have examined HSD in CALD or migrant communities [43–49]. Socio-cultural and religious factors (taboos) have been identified as contributing to attitudes and openness around HIV, sexual health in general, and health-information seeking behaviours [46–48]. A fear of stigma and social isolation/ostracism together with limited health literacy, perceptions of low risk, and perceived ‘silence’ in the broader community, as well as fear of deportation as a result of positive HIV status have also been identified as key barriers to HIV prevention, testing, and treatment [43–50].

To our knowledge only two studies have examined HSD from the perspective of PLHIV from CALD communities in Australia [50–52]. In a study which focused on migrants living with HIV in Sydney, Australia, Körner found that fear of stigma impacted disclosure decisions, which were also influenced by gender, sexual orientation, and cultural background [52]. Fear of transmission and HSD also resulted in social isolation from cultural communities, intensifying difficulties with social integration in the mainstream culture in Australia [50]. HSD was also found to be a barrier to accessing health care and support services and a major source of uncertainty regarding the prospect of staying in Australia for those living on temporary visas [51].

Focusing on HSD in CALD communities in Australia is important for several reasons not least because people born outside Australia make up approximately 30% of HIV notifications by heterosexual transmission and constitute a priority population in the latest National HIV Strategy [3]. Moreover, ethnic minorities in Australia are often grappling with different cultural understandings of HIV in resettlement countries and are more likely to be experiencing stigma and discrimination across other intersectional identities, which can compound to impact negatively on health.

### Research aims

We build on this small body of research to take an intersectional view of HSD for people from CALD backgrounds in Australia. Such an approach takes account of how the various aspects of an individual’s identity or background (e.g. gender, race/ethnicity, sexual orientation, religion, immigration status, socio-economic status) can be subject to stigmatisation which have a range of consequences for health [53–55]. The health impacts of stigma experienced at multiple locations are complex, producing significant risks and vulnerabilities [56]. We draw on Parker and Aggleton’s [57] conceptualisation of intersecting stigma in relation to HIV, which views stigma as a series of social processes that are grounded in unequal power relations and manifest through the reproduction of inequality and exclusion at an individual, interactional and structural level. In doing so, we identify the key social factors that foster and contribute to HSD for PLHIV from CALD backgrounds in Australia, and the impacts on health. The specific research questions were: 1) what were the experiences of, and responses to, HSD by people from CALD backgrounds? 2) how did these experiences of HSD intersect with other categories such as gender, ethnicity/race, sexual orientation, and immigration status; 3) what were the health impacts of these experiences of stigma/discrimination?

## METHODS

### Procedure

Ethics approval was obtained from the Flinders University Human Ethics Committee and the Royal Adelaide Hospital Human Research Ethics Committee, and all participants gave informed consent. The researchers were particularly mindful of potential issues of coercion and informed consent, power imbalances between researchers and participants and issues of language (documentation was translated into key languages and interpreters made available). In addition, given the highly sensitive nature of the research, particular attention was paid to issues of confidentiality - with verbal consent available, participants offered their choice of interpreters, interviews conducted in a place of the participants’ choosing, and all transcripts deidentified immediately and securely stored. The project was conducted in partnership with a not-for-profit organisation with a program working with PLHIV from CALD backgrounds, and ethics approval was also obtained from that organisation.

Participants were: PLHIV from a CALD background aged over 18, currently residing in SA (*N=*10), recruited through one of the major service provider organisations; CALD community leaders (*N=*14), recruited through community networks across the key African settlement communities in SA and snowball sampling; and staff from a range of service provider organisations (both CALD focused and mainstream) (*N=*50), who were recruited by approaching the key service providers in SA. PLHIV and community leaders took part in one-on-one semi-structured interviews and staff from service provider organisations took part in one-on-one semi-structured interviews and/or focus group discussions. The interviews with PLHIV and community leaders took place at a venue of the participants’ choosing and lasted on average 44 minutes and from 14 minutes up to 104 minutes. Four PLHIV requested the use of an interpreter. The focus group discussions and interviews with staff from service provider organisations took place in the offices of the organisation and lasted on average 65 minutes and from 24 minutes up to 108 minutes.

### Data collection

For the interviews with PLHIV, questions covered a range of topics including diagnosis, experiences of service use and barriers/facilitators to service use, and the impact of HIV and associated stigma on their life. Interviews with CALD community leaders included questions relating to the broader awareness of HIV in their specific community, the presence and nature of HSD and intersecting elements such as gender, religion, sexual orientation and/or immigration status, the impacts on health and their views on addressing HSD in their communities. The interviews and focus group discussions with staff from service provider organisations similarly covered topics such as the presence, nature and health impacts of HSD within the CALD communities that they work with (see Appendix 1).

Interviews were conducted by two female researchers (AZ and GR), with one from a European CALD background (who interviewed the community leaders and PLHIV and two service providers) and the other from an Anglo background (who interviewed service providers). Another researcher from an African background (LM) who was going to be involved in data collection was removed from all study materials, did not collect data, and did not have access the raw data due to concerns from members of the African community about her potential recognition of people living with HIV in their community.

### Data analysis

All interview and focus group data were transcribed verbatim and thematically analysed using the 5 stage framework approach [58]: familiarisation with the data (reading and transcripts), development of a thematic framework (done inductively and iteratively from the data), indexing (coding with NVivo Version 10 (QSR International; 2012), charting (thematic matrices charting each participant against the emergent themes), and mapping and interpretation (experiences of HSD and others forms of stigma and discrimination, responses and health impacts are outlined). Transcripts were coded by two researchers (GR and MB) and the charting, mapping and interpretation was undertaken by two researchers (AZ and MW). Findings were triangulated between the different participant groups, we undertook an expert and member checking process through presentation of findings to study participants and experts, and we undertook a negative case analysis process (which found remarkably consistent findings, with only two instances identified as outliers – reported below).

## RESULTS

Ten people from a CALD background living with HIV took part in an interview. All of these participants were migrants, of which eight were from Africa, one from Asia and one from Europe. Eight identified as female, and eight had a refugee background (Table 1). Four had been in Australia for less than 10 years and two for more than 10 (4 unknown), with an average of seven years. Six reported their religion as Christian and four did not discuss their religion. Three lived alone, four with children and three with other family or in shared accommodation.

**Table 1:** Participant characteristics

	PLHIV ***N=***10	Community Leaders ***N=***14	Service Providers ***N=***50
**Gender**			
Female	8	7	35
Male	2	7	14
Non-binary			1
**Region**			
Africa	7	14	
Asia	2		
Europe	1		
**Time in Australia**			
< 10 years	4		
>10 years	2		
Average	7 years		
Unknown	4		
**Religion**			
Christian	6		
Unknown	4		
**Living arrangements**			
With children	4		
Alone	3		
With other family	1		
Shared/supported accommodation	2		
**Service**			
CALD- focused			12
Mainstream			38

The 14 CALD community leaders were all originally from Africa and comprised seven women and seven men. 50 staff (35 women, 14 men, one gender non-binary) across 10 CALD-focused and mainstream organisations/teams took part in seven focus groups and four individual interviews. The range of organisations included multicultural services (education and empowerment), blood borne virus support services, and sexual health services. The staff from these organisations comprised clinicians (doctors and nurses), social workers, counsellors and other support workers. Around half of the staff from these services came from a CALD background and ranged in experience from long term work in HIV to relatively new to the field.

The following sections outline key themes drawn from the analysis related to: 1) experiences and responses of people from CALD backgrounds to HSD and intersections with existing mechanisms of stigma/discrimination namely gender, sexual orientation, religion and culture, as well as immigration status; 2) the health impacts of these experiences of stigma/discrimination examined through this intersectional lens.

### HSD in CALD communities

*“It is not an acceptable disease” (PLHIV, female, Africa)*

There was general consensus amongst the three participant groups that HSD was highly prevalent in CALD communities, associated with fear of transmission and death and fear of moral judgement or social rejection, and that this was often more pronounced than in non-CALD communities. One service provider summed up the power of HSD in CALD communities:

*I work with the client who comes from multicultural background … their main concern, their main issue, is stigma and discrimination. And when they’re newly diagnosed with HIV, or they know that they are living with HIV, the main issue [ … ] is not that HIV, is that how people look at them (CALD focused service, female).*

All but two (mainstream service providers) participants believed that HIV-related stigma was more of an issue in CALD communities. A key aspect highlighted in interviews was the collectivist cultural system of many of these communities, in which bonds to family, neighbourhood and community prevail and where stigma and discrimination manifest as cultural and social occurrences through the actions of whole communities/groups. A female community leader noted the connection between individual actions and family reputation in this context:

*There is this unity in the family. Your father is having very hard time. You share this feeling, so if there is this stigma on one of the family members, the stigma goes to the whole family (community leader, female, Africa).*

Another female community leader described HIV as *“kind of a family disease”.* A woman living with HIV from Africa added to this by observing the way in which the families of PLHIV in her country of origin are “*disgraced*.”

Service providers also acknowledged the significance of collectivist cultures in the manifestation of HSD for people from CALD backgrounds, identifying the importance of collectivism to one’s identity:

*Within an individualistic society, I think it’s much easier … you’re individual, you make your own choice ‘I don’t need to be part of that group – I go find another group that I feel connected with’ and it’s okay. ‘You don’t accept me – bad luck’. But within a collective society I think, there is an ongoing challenge between the multiple identities people have - the identity of belonging to a culture that … they’re proud of, and the identity of living with HIV (CALD focused service, female).*

Another said that internalised stigma is greater in collectivist cultures *“because your identity is so vested in collective it’s so hard to separate”* (CALD focused service, female).

In addition to cultural beliefs/systems, religion formed the foundation of views on HIV in CALD communities, particularly African communities. Amongst the community leaders and PLHIV accounts was a pervasive religious belief about HIV as a punishment from God, specifically articulated in relation to female promiscuity or sex outside of marriage and homosexual activity. A young woman from Africa living with HIV had internalised this belief by seeing her HIV status as an appropriate punishment for past transgressions: *“I did a lot of wrong things, maybe God doesn’t like that way, my way, he give me this disease, and I am accept that.”*

We discuss further below how these intersections between religious and cultural beliefs underpin HSD as well as other intersecting forms of stigma associated with gender relations and sexual orientation.

### Intersecting stigma: gender, sexual orientation, immigration status and HSD

*“It’s quite a multi-layered issue” (CALD focused service, female).*

Interviews revealed an interplay between HSD and gender relations, sexual orientation, immigration status and non-mainstream cultural systems and religious beliefs, which gave rise to multiplicative experiences of HSD for those living with HIV from CALD backgrounds. A number of service providers highlighted this *“intersectionality of stigma”* (CALD focused service, female), which in many cases resulted in “*layers of oppression*” (mainstream service, female) for members of CALD communities living with HIV:

*You have someone who is living with HIV, and is gay, and then has the double stigma, and then you have someone who their experiences [ … ] of stigma and discrimination will differ completely if they had African added on top, and Muslim added on top. So each, you can say its double, triple, quadruple (CALD focused service, female).*

*If you’re woman you are most affected by stigma and discrimination and imagine if you are from a religious background - if you are Muslim [ … ] it will add to that, if you are a mother it will add to that (CALD focused service, female).*

#### Gender Relations:

Patriarchal gender relations featured in the accounts of most participants when describing HSD. As a consequence of heteronormative male power (e.g., greater sexual autonomy for heterosexual men, stigma associated with female sexual activity outside of marriage, denial of homosexuality and women’s greater responsibility for caregiving) together with other cultural practices and sexual assaults during war, women can be more vulnerable to infection. Women may also be left in the dark about the source of their infection and are thus susceptible to blame and stigma—including self-stigma—and the associated ill effects. While one male mainstream service provider noted that the disproportionate impacts of stigma and discrimination on women was universal – “*you know, which country doesn’t that happen in?” -* overall participants pointed to the particular vulnerabilities of women from CALD backgrounds.

Community leaders detailed the acceptance of polygamy in many African cultures in addition to sanctions against female promiscuity, and the moral consequences of these patriarchal and heteronormative gender relations when it comes to attributing blame for the transmission of HIV:

*For women, that she had been a prostitute – she’s going out with other mens, even if she got [HIV] from husband, everyone, including men and women will be looking the woman as the main cause of the problem (community leader, male, Africa)*

Interestingly, while the male community leaders acknowledged the impact of patriarchal and heteronormative gender relations on blame and potential stigma associated with HIV, they did not offer any judgement. In contrast, a female community leader offered a more critical reflection:

*[In Africa] men can have more than one relationship, up to four wives, which is unfair! The women can’t – even two. So, having two means immoral. And you deserve God’s punishment (community leader, female, Africa).*

Service providers also identified these gender relations, with all agreeing that women suffer particular blame and judgement:

*There’s also the stigma attached to the way it’s contracted [ … ]. You know, you’re some sort of woman that [ … ] even though they may have been raped or transmitted by males, or whatever, that’s beside the point, but the label that they get with contracting it, promiscuity and things like that (CALD focused service, female).*

A young woman from Africa living with HIV, mirrored this perspective:

*When you get HIV, no one cares if it is even your fault, if it’s your boyfriend, it just looks like you have sinned, you’re prostitute, you have been sleeping around (PLHIV, female, Africa).*

These quotes highlight a moral emphasis on women’s sexual practices and the presence of patriarchal and heteronormative gender relations in terms of how judgements around transmission are almost always unfavourable towards women. However, while men enjoy disproportionate power in many CALD communities, male sexual practices that fall outside of cultural norms of heterosexuality are also highly stigmatised.

#### Homosexuality:

Heteronormative gender relations were similarly at play in the participants’ accounts of homosexuality, which was highlighted as a key intersecting social category that amplified and complicated HSD. Participants reported that generally speaking, homosexuality for people from CALD backgrounds is not acknowledged as existing or is simply not spoken about. Service providers from mainstream organisations noted the denial of homosexuality in a range of CALD communities, in particular Middle Eastern, African, Asian and South American cultures – *“they just say they [ … ] don’t have men that have sex with men in their community”* (mainstream service, female)

Community leaders also acknowledged the silence surrounding homosexuality in most African communities: *“[it’s] one of those issue which not talked about at all”* (male) and the religious and cultural beliefs contributing to the taboo nature of homosexuality in their community *“we think that they are sinful* (female)” – and the impact on community members:

*If it is known [homosexual practices/orientation] he could be or she could be probably maybe even discriminated even more [than] someone with HIV [they would have to] go to live in the country, you know, yeah. That would be terrible (community leader, male, Africa).*

Service providers further identified religious beliefs and homosexuality as complicating factors for men who have sex with men (MSM) from CALD backgrounds in addition to their HIV status:

*So quite a few with the MSM from CALD backgrounds … their issues around not being out and being homosexual is probably even as important, if not more important [ … ] I think that is definitely also complicated by - religion is a big part of things as well (mainstream service, female).*

As these excerpts, from across the range of participants suggest, HSD and sexual orientation—underpinned by culture, religion and heteronormative gender relations —intersect to produce a compounding risk for CALD community members living with HIV of being socially isolated.

#### Immigration status:

For many people from CALD communities, HSD intersected with immigration status in two ways: through fear associated with the impact of a positive HIV diagnosis on visa and immigration aspirations; and resettlement factors for those from refugee backgrounds (e.g., existing social isolation, poverty, trauma and mental health issues and so on). Individuals on temporary refugee visas, for instance, who are HIV positive were identified as being particularly fearful of potentially discriminatory immigration practices and fear of being deported back to their countries of origin. A service provider told of a Middle Eastern client who became agitated during a counselling session as he was convinced an air conditioning duct housed a hidden camera linked to immigration:

*That level of fear and trepidation about [being potentially monitored by immigration]. I said, ‘what frightens you about this?’ He said ‘if the Australian government finds out that I’m gay and I’m HIV positive they’ll deport me back to [country] where I’ll be executed (mainstream service, female).*

Other service providers shared similar accounts of clients on temporary visas and fears around being deported on the basis of their HIV status taking precedence over the disease and contributing to non-disclosure:

*It’s more about ‘am I able to stay in Australia? Are you going to report me?’ or anything like that so it’s not really about the disease for some people (mainstream service, male).*

Broader settlement issues were also seen to complicate HSD particularly for newly arrived women from refugee backgrounds, often single parents who were offered “*very fractured or very limited settlement support services”* (CALD focused service, female).

Other service providers also spoke about international students and migrant workers who wished to apply for permanent residency, which requires them to be tested for a range of illnesses, including HIV- at which point an “*HIV diagnosis sometimes comes up [ … ] and that’s when everything just falls apart”* (mainstream service, female). Service providers talked at length about clients who are engaging in homosexual relations which are taboo or even outlawed in their countries of origin and where there is no reliable treatment for HIV as outlined by a service provider working in a mainstream service:

*I’ve had patients in an application process, you know, it’s been extremely stressful for them with how long and drawn out the process is of working out whether you’re going to be sent back to a country where you won’t be able to get any treatment and antiretrovirals and your life expectancy would be four or five years versus here where you can have a long, full life (mainstream service, female)*

These intersecting and overlapping aspects of HSD have significant impacts on PLHIV. In this next section we explore the health impacts in more detail.

### Impact of stigma on health and wellbeing

*It’s not actually HIV that is killing them, it’s because the stigma that the community have against them* (CALD focused service, female).

Participants identified a number of negative impacts on both mental and physical health of HSD – all of these were associated with fear of HIV positive status disclosure leading to social isolation, which was overwhelmingly identified as having a more detrimental impact on the wellbeing of CALD community members as members of collectivist cultures. As the key pathway to worse health outcomes, fear of disclosure was associated with the vigilance required to manage risk of disclosure and subsequent social isolation, leading to delayed testing and diagnosis, limits to accessing health services for treatment and/or counselling and impacts on treatment adherence - alongside feelings of shame and self-blame. The following sections report on these health impacts through the lens of intersecting stigma highlighted above.

#### Fear of disclosure - social isolation:

A fear of disclosure and anticipated (and actual) social isolation was reported as the foundation of the detrimental impacts of HSD. This was relevant both in terms of a fear of being socially isolated if their HIV status was disclosed (stress, burden of carrying a secret, avoidance of certain people and places) as well as the actual impact of social isolation/ostracism from friends and community and self-isolation on health (absence of support, loss of identity, loneliness etc). Compelling accounts of fear of disclosure and social isolation came from all participants and were located at the intersections of HSD, gender, sexual orientation, collectivist culture and religion*.*

Service providers detailed their CALD clients’ preference for secrecy around HIV. For example, a male working in a mainstream service said:

*[P] eople can’t be out about their HIV status to almost anyone [ … ] what I’ve seen in almost every person is that real and intense fear of being ostracised from the community, from their own community (mainstream service, male).*

New arrivals who have not yet established social relationships with their cultural community can also find themselves fearful of making connections in case their HIV status and in some cases their sexual orientation is disclosed:

*There is one guy in particular that I’m thinking of [ … ] he came and got diagnosed with HIV in Australia and doesn’t have any support networks in Australia and is really fearful to connect with his community here because of his HIV status and his sexuality (CALD focused service, female).*

Several participants living with HIV revealed how they had isolated themselves in order to avoid disclosure:

*My family don’t know anything. Even not family, even the community, I don’t want to tell anything because this thing is very hard if someone know about this. No one accept anything. No one treat you like good thing, no one give you good hug or something, (PLHIV, female, Africa).*

*Because I am still living in fear. So much fear [ … ] I think I’ve been living just myself – I’ve been inside [ … ] I was so scared – like the African community – if they get to know, they won’t talk to me. I felt better inside the house [ … ] I just wanted to be on my own (PLHIV, female, Africa).*

Community leaders likewise pointed to this fear but also the reality of social isolation for those whose HIV status became known:

*I have some example within the community that happens [ … ] where both male and female were completely you know excommunicated from the community and they were seen as people who are really bad (community leader, male, Africa).*

Service providers spoke about the health impacts of maintaining vigilance around the disclosure of their HIV status for women, particularly those with children:

*Imagine someone living double life and trying to hide, their health, so you can see two different personality, someone trying to be very strong, trying to do lots of hard work for them to survive, in their own world. There is that other side where they’re totally scared [then] depression will start to kick in, high level of anxiety as well, and fear, and so you can imagine what their mental health status would look like (CALD focused service, female).*

Vigilance in protecting one’s HIV status from being disclosed was also referenced by other service providers, who described the ill effects as “*very, very profound, profound isolation”* (CALD focused service, female) and *“a sense of alienation because of their internalised stigma”* (CALD focused service, female).

Such social isolation whether self-imposed or stemming from the actions of others was seen as damaging for mental health, particularly in the context of collectivist cultures, as evidenced by the following excerpts from community leaders:

*Of course, isolation give people depression … and the fact that they can’t talk to someone about it, they just keeping it to themselves, it’s a big load to carry. And if you stepped out of that to share it with someone, you set yourself up to be discriminated (community leader, female, Africa).*

*It’s because you have this disease you are excommunicated. You are not having any friend... Become very hard. So this is really putting a lot more health problem apart from the one [HIV] that [you already have] (community leader, male, Africa).*

Those living with HIV likewise highlighted the negative health impacts of social isolation. For example, one woman shared:

*When go to work, really shy with myself. Not want to eat with friend, not wanna walk near, not want nobody know I’m sick in there. [ … ] even walking with people, [crying] I tell myself, no I have to get a little bit far away, because I’m sick [crying]. Even to go eat, I cannot (PLHIV, female, Asia).*

In contrast, a woman living with HIV from Africa revealed that through keeping her positive status a secret, she was able to maintain close ties with her community and a sense of hope for the future, though her view that certain death would result from disclosure reveals the burden of fear she carries:

*If I explain to anyone, I am sure gonna affect my – affected me – I’m gonna be die soon. The main thing for a human, it is hope and mentally healthy. I know if I share with anyone, other people, I will affected me. Symptom have to keep it for myself. And work like other normal person. Improve my life. That’s it (PLHIV, female, Africa).*

Underpinning this fear of disclosure and social isolation was internalised stigma and feelings of shame, which were seen to directly negatively affect mental health, particularly for women.

Female community leaders further identified shame as a key affective response to HSD for PLHIV: “*That person feel down, depressed, ashamed, guilty, there is shame, there is guilt.”*

Service providers also noted the compounding shame and self-blame associated with experiences of sexual violence as the means of transmission for some clients:

*Because of their sense of self shame, even though the woman may be fully aware that it wasn’t her fault but it’s that self-shame also (CALD focused service, female).*

*And psychologically the ongoing feelings of shame and grief of what they’ve lost, or what they think they’ve lost in regards to relationships and community inclusion and things like that (CALD focused service, female).*

One woman from Africa living with HIV had worked hard to keep her illness secret because HIV “*is not an acceptable disease.”* However, in another part of the interview she went on to resist community judgement and shame:

*Why people they look at me like I’m a different person. Or shame on me or – it shouldn’t be like that. [ … ] You have to take care of other people. And people should see me like normal, because it is not my fault (PLHIV, female, Africa).*

#### Fear of disclosure - testing and diagnosis:

Late diagnosis and delayed treatment have significant consequences for health outcomes [59]. Participants reported that fear of HSD was a barrier to being tested, as one woman living with HIV noted:

*I also feel, out of fear, people don’t get tested because of how they will be treated, how their family will be treated. How the society will treat them. So you don’t get tested and you don’t even know you have these things (PLHIV, female, Africa).*

Service providers similarly described the impact that fear of a positive diagnosis and HSD can have on testing:

*If you’re stuck in a position where you can’t tell anyone, you can’t tell anyone your test results and you’re scared to take treatment because that could out you as well, then it’s easy to see what’s the point in being tested? Like why get tested and then I’m not going to be able to do anything about it because I’m too scared of the ramifications (mainstream service, male).*

This included for those who wished to apply for citizenship:

*If you get positive because it’s an immigration requirement so people who haven’t got their citizenship wouldn’t want to disclose. Although they would be tested but they wouldn’t want to go for tested (community leader, male, Africa).*

Service providers also identified fear of being seen even being tested as a barrier, regardless of the result: “*If someone sees me go in, and they find out, they will automatically assume – just getting tested for it - you are assumed to have it. Yeah, so there’s that stigma by association”* (CALD focused service, female).

Stigma associated with homosexuality and HIV was also a health concern due to men not undergoing testing despite engaging in ‘unsafe’ sex, which has ongoing implications for treatment as well as the transmission of HIV. A mainstream service provider explained:

*They may be married. They may be having sex with other men on Grindr and various other apps. They may well be having unsafe sex and they may well actually have given their wives HIV. Because none of them are testing we won’t know for at least another five, six, seven years where there are late presenters with actually an AIDS defining illness (mainstream service, male).*

#### Fear of disclosure- service access:

Fear of disclosure, through being ‘seen’ accessing services was reported by community leaders: *“if I go there* [HIV service]*, someone may see me going in and they’ll know that I’ve got it”* (community leader, female, Africa).

Service providers identified similar concerns in accessing services:

*The waiting room is an area of great tension for some patients … On occasions patients, I haven’t been able to find them because [ … ] they’ve moved off somewhere more private, obviously to avoid feeling like their status might have been disclosed by being seen in the waiting room (mainstream service, female).*

Service providers and community leaders also talked about HSD barriers to other service aspects including reluctance to link in to a support person or mentor from their community: “*but there’s absolute concrete resistance to that, like ‘no, I will not do that”* (mainstream service, male) and taking information *“none of my clients will take any brochure or any leaflet or any information”* (CALD focused service, female).

These barriers intersected with fear of disclosure of sexual orientation or sexual activity for MSM and a concern about attending support services for gay men: *“African men are unlikely to come out as gay currently so they’re unlikely to actually come to a service like ours to go and have an STI/HIV test”* (mainstream service, male). A community leader also noted barriers to attendance at information sessions about homosexuality and HIV if he was to arrange them: *“I won’t even get single person to this session”*.

#### Fear of disclosure- use of interpreters

The use of interpreters was a key element of service access that was a concern in terms of fear of disclosure. A central issue was a fear of clients about breeches of trust by interpreters revealing HIV status to other community members - as one community leader put it: “*we not trust interpreters to bring it back to the community [ … ] this community is smaller. And if it goes out of one person, everybody knows about it”* (community leader, male, Africa). A woman from Africa living with HIV likewise reflected on this risk:

*Some interpreters ‘no good’ [ … ], if I tell for you, you tell for your friends, then she tell another person, then every people she know that thing. And I know her in church, sometimes if I go ‘oh this lady she have this thing, and because of that now, if I go everywhere I say ‘no I don’t want an interpreter’ (PLHIV, female, Africa).*

Service providers similarly noted fear around interpreters disclosing HIV positive status:

*There are whispers in the community again, now, in one of the African communities about a recent possible disclosure that the community thinks came from a translator with a medical service (mainstream service, female).*

*I’ve had [a client with HIV] pull me down our back corridor and say ‘that person goes to my church’ You know, they’ve instantly left the waiting room and said ‘you’ve got to get rid of - that person can’t see me here’ (mainstream service, female).*

Service providers reported that HIV positive clients managed this risk by using telephone rather than face-to-face interpreting and requesting interpreters from interstate or from different countries even if their language proficiency was more limited. However, for newer language groups interpreters were often from the same community and given the small size of the communities, interpreters could still potentially recognise voices or other details about the client. Another strategy used was developing a relationship with a trusted interpreter, though this was not without complications, as it was not always possible to request the preferred interpreter.

Notwithstanding fears around disclosure, service providers also noted that the negative reactions of some interpreters to medical information in consultations have caused distress for PLHIV including an example of phone interpreters hanging up once HIV is mentioned:

*Sometimes I’ve had that happen twice in the one consultation with two different interpreters and it’s really upsetting for the patient because they’re being judged...stigmatised by some interpreter on the end of the phone (mainstream service, female).*

#### Fear of disclosure- treatment adherence:

Service providers reported that treatment adherence was also compromised due to fear of disclosure of a HIV positive status, likely impacting negatively on health outcomes:

*People, they prefer to die from HIV, not to get treated, just for people not to know, not to be isolated. They choose to die, not to take medication, but not to be isolated and stigmatised (CALD focused service, female).*

In particular, service providers highlighted concerns of their patients about medications potentially revealing their illness:

*If you can’t tell anyone else in your family why you’re taking that medication every day, so hiding your medication so nobody knows, explaining why you have to go to doctor’s appointments or hospital [ … ], so I guess in terms of their medical care it [stigma] can impact (CALD focused service, female).*

HSD also acted as a barrier to accessing subsidised medicines. One community leader described a pharmacist that offered subsidies for HIV medication: “*But then some people will be afraid of going there because they think they will be singled out”* (community leader, female, Africa). A medical practitioner also gave an example of someone who had a life-threatening allergy to one of the HIV medications but would not carry an allergy card because it had the potential to reveal their HIV positive status. Other service providers reported that others ceased treatment while overseas fearing the medication may disclose their HIV positive status:

*I think there are difficulties when they go back to their countries of origin for holidays [ … ] I have patients who have been virally suppressed, gone overseas, come back and their viral load is through the roof [so] most definitely there are issues around medication, taking medication, being seen to take medication (mainstream service, female).*

## DISCUSSION

In a 2017 address, UNAIDS International Goodwill Ambassador Kenneth Cole noted “it has been said that stigma has killed more people than the HIV virus” [60] - and the findings of this paper likewise indicates the health risks of HSD for people living with HIV from CALD backgrounds. The analysis revealed an overwhelming sense of the weight of HSD on CALD community members and this was perceived as greater than for those from non-CALD backgrounds. HSD played out at the key intersections of gender, sexual orientation and visa status, overlayed by religion and culture. HSD was identified as a key mental and physical health risk by all participants, with pathways identified through fear of disclosure leading to social isolation, delayed testing, service access barriers, compromised treatment, and lack of social support.

In relation to experiences of, and responses to, HSD by people from CALD backgrounds, this study revealed significant experiences of felt, enacted and internalised stigma for PLHIV from CALD backgrounds, and their families and communities – extending even to concerns about the involvement of a researcher from an African background in the study. Much of the stigma can be attributed to misconceptions associated with HIV that are influenced by factors such as ethnicity, gender relations, and religion originating from home countries with collectivist cultural systems [21–23]. The paper’s findings reflect an existing international literature on the prevalence of HSD in general [12] and in CALD communities specifically [28–31]. Previous research has likewise identified HIV as highly stigmatised and particularly so in CALD communities. While there is some indication of reductions in HSD in general, this may not be uniform across communities [9]. With all but a couple of exceptions (of two non-CALD service providers), participants in our study identified HSD as a bigger issue for CALD communities than the general population. In particular the impact of stigma in a collectivist culture, where the actions of an individual are often intricately linked to their family and broader community, [52] was highlighted, and this was seen as magnified in the migration context where migrant communities were often small.

Less explored in these previous studies is a consideration of intersectionality [53–55]. In relation to the ways in which experiences of HSD intersected with other categories such as gender, ethnicity/race, sexual orientation, and immigration status, our study found HSD intersected significantly with a range of other social categories and stigmatised identities and that these intersections were essential to understanding the experience of HSD in CALD communities [36, 37, 42]. Overlaying many of the combined stigmas was religion. Religion is an important part of individual and community life for many people from CALD backgrounds [61] and prescriptions around sexuality and gender relations was seen by participants as influencing how HSD was experienced. In particular patriarchal and heteronormative gender relations were highlighted by all of the participant groups as relevant to the experience of HSD. Overwhelmingly women living with HIV were seen as more at risk of being the target of HSD – often wrongly blamed for disease transmission and perceived as having compromised sexual morals. Women reportedly internalised these views and, in many cases, isolated themselves. Heteronormativity was also relevant to a consideration of the relationship between sexual orientation and HSD. The association of HIV with homosexuality and sexual ‘deviance’ by most religions was felt significantly by men who had sex with men who were HIV positive, whether or not they identified as homosexual. The study also found that HSD compounded the vulnerability felt by people on temporary immigration visas.

A body of research has highlighted the impact of HSD on mental and physical health [10, 24, 25, 42, 62] including for those from CALD backgrounds [36, 37]. These findings were reflected in our study and in relation to the health impacts of experiences of HSD the analysis found significant potential impacts for both mental and physical health that appeared heightened for people from CALD communities, particularly those from collectivist communities. A fear of disclosure of one’s HIV positive status constituted the key pathway between HSD and poorer health outcomes. In terms of mental health this manifested through social isolation (self and imposed) and a lack of social support from family members, friends, dwelling community and caregivers, because of the taboo nature of HIV, alongside stress and internalised negative views of self, mirroring previous studies more generally [7, 42, 63, 64]. Social connections and support, particularly in diaspora communities, are known to be key mental health promoters, so self or imposed social isolation limits access to these beneficial resources. Likewise, negative self-concept has also been linked to poorer mental health outcomes [55, 65].

Physical health outcomes were also seen to be detrimentally impacted by HSD. HSD was linked to delayed testing which has been identified as key issue for PLHIV from CALD backgrounds [3] and linked to worse health outcomes [66, 67]. Likewise fear of disclosure through being seen at services or concerns about interpreters or taking medication, compromised access to treatment and treatment adherence - both of which have been associated with worse health outcomes [43, 45–47, 68].

The intersections identified in the study are important for considering the health impacts of HSD. As Aggleton and Parker’s model highlights, HSD sits alongside other forms of inequity and oppression. In this way, not only may some groups be more susceptible to HSD and its detrimental effects, they are likely already experiencing other forms of stigma and discrimination through racism, sexism and homophobia that make them more vulnerable to health inequities in the first place. Addressing HSD in CALD (and other) communities therefore requires an engagement with these overlapping forms of inequity.

In this paper we have examined the perspectives of community leaders and service providers from both CALD and non-CALD focused services covering all the key providers in South Australia, as well as PLHIV from CALD backgrounds about which very little is known. The in-depth qualitative data enabled an analysis of these multiple voices and the intricate way that HSD intersected with a range of other elements of people’s lives. However, there was a small number of PLHIV, largely from Africa, and the community leaders were all from Africa. Given that cultural systems have a bearing on how communities respond to HIV and the ways in which HSD plays out, it is likely that cultural factors and personal experiences will vary between individuals and communities and further research that involves PLHIV and community leaders from a more diverse range of backgrounds is required to provide more insight into this. We did not have representation of all of the intersecting identity characteristics that emerged as relevant in the analysis amongst our PLHIV participants. However, the triangulation with service provider and community leader perspectives provided additional input into understandings of this, and the consensus across the participant groups around HIV as a stigmatising condition was striking. There are of course a range of broader social and economic costs associated with HIV in resettlement countries, particularly as they relate to lost productivity and total medical costs across the life of the illness [69]. While beyond the scope of this paper, these impacts likely compound and are compounded by HSD, which warrant further research.

## Conclusion

The focus of this paper was on the experiences of, and responses to, HSD by people from CALD backgrounds, and the potential health impacts of these experiences and responses. The paper makes a unique contribution to the literature, through canvassing the views of multiple participant groups and drawing on the lens of intersectionality to highlight the complex intersecting character of HSD in CALD communities. It further describes the range of pathways through which HSD can affect mental and physical health for PLHIV from CALD backgrounds and the communities in which they live.

In addition to addressing the intersecting inequities highlighted by Parker and Aggleton [57], key policy mechanisms to address stigma need to be co-designed and implemented with PLHIV from CALD backgrounds, and research is required to evaluate these approaches. This includes sufficiently funded culturally appropriate services for CALD people, carefully established support groups, culturally appropriate education and information, the involvement of CALD community (and religious) leaders, HIV positive speakers from CALD communities, training for interpreters, and addressing policy and legislative barriers for example for those with temporary visas. The impact of HSD on the health of people from CALD backgrounds is such that this work must be tackled with the same urgency as vaccine trials and treatment development.

## Supplementary Information

**Additional file 1: Appendix 1**: Interview guides.

## Data Availability

The datasets generated and/or analysed during the current study are not available (publicly or privately) due to the highly sensitive nature of the interview material.

## Abbreviations

CALD: Culturally and Linguistically Diverse
HSD: HIV-related stigma and discrimination
MSM: Men who have sex with men
PLHIV: People living with HIV
