# Supplementary material for: “It is not an acceptable disease”: A qualitative study of HIV-related stigma and discrimination and impacts on health and wellbeing for people from ethnically diverse backgrounds in Australia
Source: BMC Public Health. 2021 Apr 23;21:779. doi: 10.1186/s12889-021-10679-y (PMC8063420; doi:10.1186/s12889-021-10679-y)
Supplement: Supplementary file 1 — Additional file 1: Appendix 1: Interview guides. [file 12889_2021_10679_MOESM1_ESM.docx]

**Appendix 1: Interview guides**

**People living with HIV**

Diagnosis:

- How long living with HIV?
- How has this been?
- Do many people know of diagnosis?

Service use:

- what services used – both HIV specific and others
- experiences of
- barriers and facilitators to use

Impact [of HIV and stigma] on life:

- Health and wellbeing
- Relationships
- Social networks – including specific ethnic community
- Family
- Other – eg. education, employment, housing

Anything specific about being from a CALD background that they think is relevant to experiences of living with HIV?

**[prompt - stigma experienced/in CALD communities]**

**Community Leaders**

Background:

- How long in Australia
- Which community/s do you see yourself belonging to (check which community they are referring to throughout the interview – ie broader ‘African’ or specific country or other)
- Role in the/those communities
- Any involvement in HIV prevention or awareness activities

Views of your community about HIV:

- Any stigma – stories/examples of where you hear this, what do people say and where
- Differences with general/mainstream community views vs. own cultural group – more or less or different stigma

HIV stigma:

- Impacts of someone’s HIV status being known in the community
- Reluctance to be tested and attend treatment services and medication adherence – examples if you have seen or heard of this happening
- Direct discrimination
- Social relationships – of disclosing or not disclosing status
- Relevance of mode of transmission (e.g. needles/drug use, blood transfusion, heterosexual or homosexual sex)

Health and wellbeing impact of stigma and issues above relating to this:

- the individual involved – treatment, stress, coping mechanisms
- their family and friends
- the broader community (own community) (ie community coherence and harmony) – how does the community deal with it

Are there any other factors in people’s lives that also impact on how they experience living with HIV and any stigma associated with it (do these make it easier or harder or different – examples/stories):

- Refugee status
- Gender
- Sexual orientation
- Tribal or ethnic differences
- Any other factors you think are important

Addressing stigma:

- Do you have any suggestions for addressing HIV stigma both in your own community?
- Any involvement in trying to address HIV – what has been successful and what not so successful
- Aware of any other ways the community is dealing with it?
- Key barriers to addressing stigma and discrimination

**Service providers**

- What are your roles in working with people with HIV from CALD backgrounds?
  - Own background/role in the communities?
- What has been your experience of witnessing HIV related stigma in these communities?
  - How does it differ to non-CALD communities?
- What do you think the impact of this stigma is on people?
  - Testing and access to services
  - Social relationships/inclusion
  - Health and wellbeing
  - Impacts on family members
  - Cultural impact on the community
  - Other impacts including employment, self esteem
- Are some people in the community more vulnerable to this – eg. women, sexual orientation, ethnicity, refugee status?
- What is already being done to address stigma and HIV
  - what has worked and what hasn’t
  - why – ie facilitators and barriers
  - community reactions/priorities
- What would you like to see happen to stigma in CALD and broader communities?
  - Barriers/facilitators to doing this in particular communities?
  - Different to non-CALD communities?
- Key issues facing CALD clients living with HIV
